# THE RELATIONSHIP OF AGEISM, INTENTION TO WORK WITH OLDER ADULTS, AND SOCIAL DESIRABILITY

**DOI:** 10.1093/geroni/igac059.2442

**Published:** 2022-12-20

**Authors:** Maria MacLean, Jessica Strong

**Affiliations:** University of Prince Edward Island, Charlottetown, Prince Edward Island, Canada; University of Prince Edward Island, Charlottetown, Prince Edward Island, Canada

## Abstract

Previous research demonstrates: 1) men and younger adults have higher negative ageism scores than women and older adults 2) higher scores of negative ageism are associated with lower intention to work with older adults and 3) women and older adults have higher scores for social desirability. It remains unclear how these factors interact. University students (N=547) aged 16 - 59 (Mean = 20.6) completed a survey measuring positive and negative attitudes towards older adults, intention to work with older adults, and social desirability. ANOVAs found a significant effects in negative ageism based on age, F(1, 3) = 6.69, p = 0.01, ω2 = 0.01, and gender, F(1, 3) = 11.43, p = 0.001, ω2 = 0.02, with a small effect size, but no significant interaction between age and gender. Young adults (M = 22.3) and males (M = 21.5) demonstrated more negative ageism than middle aged adults (M = 23.1) and females (M = 22.7) (lower scores indicate negative attitudes). An ANOVA of gender x age x social desirability was also significant for negative ageism, F(11) = 2.00, p = 0.03. However, there were no significant effects or interactions for gender or age on positive ageism and intention to work with older adults, or when social desirability was added. Although there were differences between demographic and social desirability groups for negative ageism, this relationship was not found for positive ageism. We expected social desirability to play a role in ageism, but this was not the case in the current sample.

